# Editorial: A lifecourse perspective on polycystic ovary syndrome (PCOS): bridging gaps in research and practice

**DOI:** 10.3389/fendo.2026.1794443

**Published:** 2026-02-05

**Authors:** Anagha Joshi, Mahnaz Bahri Khomami

**Affiliations:** 1 Department of Clinical Science, Computational Biology Unit, University of Bergen, Bergen, Norway; 2 Department of Biotechnology, Bhupat and Jyoti Mehta School of Biosciences, IIT Madras, Chennai, India; 3 Monash Centre for Health Research and Implementation (MCHRI), Monash University, Melbourne, VIC, Australia

**Keywords:** disease, endocrinopathy, life- course perspective, PCOS (polycystic ovarian syndrome), biomarkers, epidemiology

Polycystic Ovary Syndrome (PCOS) is one of the most common endocrinopathies affecting ovarian function, with an estimated 116 million women impacted worldwide. It is clinically defined by ovulatory dysfunction, hyperandrogenism, and polycystic ovarian morphology (PCOM) or elevated anti-Mullerian hormone (AMH), though there is a wide variation in symptoms and trajectories across life. Despite the knowledge of multisystemic health complications spanning reproductive, metabolic and psychological aspects, clinical interventions often narrowly focus on reproductive health alone, inadvertently neglecting the full spectrum of this multifaceted condition. This Research Topic has gathered state-of-art research in PCOS focusing on some of the key aspects of PCOS as well as co-morbidities associated with PCOS, using diverse research approaches ranging from epidemiological, molecular to clinical analyses of PCOS. This Research Topic is a small step towards creating a more detailed multidimensional picture of PCOS as a disorder, building on its multisystemic health complications across endocrinology, metabolism, immunity, mitochondrial function, circadian biology, and psychological aspects.

## Epidemiology of PCOS

This Research Topic documents, through epidemiological analyses, rising global PCOS burden among adolescents and young adults with projections to 2036 to support public-health planning of life-course prevention strategies (Wang et al.). PCOS remains underdiagnosed for many reasons, including its wide−ranging symptoms, which are often treated separately rather than being recognized as part of a single condition. Digital PCOS cohorts may provide scalable remote phenotyping to map health burdens and lifestyle characteristics across the life course in both diagnosed and possible PCOS. Thus, bridging clinic-based cohorts and broader community samples will be first step towards understanding the true diversity of this condition, beyond strict clinical definitions (Peebles et al.).

Hormonal dysregulation is at the heart of PCOS, where excess ovarian and adrenal androgens drive impaired folliculogenesis, leading to ovulatory dysfunction and clinical symptoms such as hirsutism and acne. AMH is typically elevated in PCOS as higher AMH is associated with increased follicular count. The use of AMH as a diagnostic and prognostic biomarker continues to be debated in PCOS research and clinical practice (Ran et al.), although the 2023 International PCOS Guideline supports its use for defining PCOM in adults as an alternative to pelvic ultrasound, with either AMH or ultrasound used to avoid overdiagnosis.

Insulin resistance and metabolic dysfunction form a core aspect of PCOS and are extensively explored in this Research Topic. Intrinsic insulin resistance is thought to amplify hyperandrogenism via ovarian theca cell stimulation, leading to glucose intolerance and dyslipidemia. Mitochondrial DNA oxidation and altered mtDNA content vary across metabolic PCOS phenotypes, linking mitochondrial oxidative damage to systemic metabolic dysfunction (Rojo et al.). Furthermore, genetic analyses of mitochondrial tRNA mutations have explored maternal inheritance patterns that may contribute to familial clustering and phenotypic heterogeneity in PCOS (Nawaz et al.).

Simple anthropometric measures remain clinically useful to estimate metabolic dysfunction. A meta-analysis found that the waist-to-height ratio correlates strongly with insulin resistance in people with PCOS, supporting its role as a rapid, low-cost screening tool to identify cardiometabolic risk in routine practice (Lan et al.). A validated nomogram for predicting insulin-resistance risk in Chinese women with PCOS is a practical clinical tool for individualized risk stratification and early intervention planning (Guo et al.).

Towards therapeutic targets, zinc sulfate showed promise by improving insulin resistance. This effect appears to be mediated by a reduction in hepatic oxidative stress and decreased apoptosis via NF-κB modulation in PCOS rat models (Kang et al).

Infertility is common in women suffering from PCOS, but it is important to recognize that PCOS represents a broader reproductive health burden that extends beyond conception alone, including increased risks of adverse pregnancy outcomes. Oral glucose tolerance test-based measures of insulin resistance were associated with embryo quality, demonstrating the correlation between reproductive and systemic metabolic dysfunction (Hu et al.). Furthermore, a multi-omics study profiling follicular fluid demonstrated metabolic alterations associated with reduced oocyte quality in PCOS (Chen et al.). A Mendelian randomization study explored causal links between fertility nutrient supplementation and PCOS risk and identified omega-3 fatty acids, berberine, and curcumin, as potentially effective for improving metabolic and reproductive abnormalities associated with PCOS (Shao et al.). However, a review of dietary supplements highlighted challenges in this field, including heterogeneous evidence and a call for higher-quality trials to define efficacy, dosing, and safety (Han et al.). Women with PCOS face additional risks during fertility treatments such as ovarian hyperstimulation syndrome (OHSS). To minimize OHSS in women with PCOS, individualized stimulation protocols and preventive strategies are needed to reduce iatrogenic harm during IVF and to balance fertility goals with safety in this high-risk group (Leathersich et al.).

Immune dysfunction is thought to play a key role in PCOS pathogenesis, as high immune cell infiltration is observed across multiple organs, representing a systematic low-grade chronic inflammation. Multi-omics analysis has identified Complement Component 3 (C3) as a central driver of immune dysregulation in PCOS, pointing to innate immune activation as a mechanistic hub connecting inflammation with ovarian and metabolic dysfunction, and further suggesting immune pathways as potential therapeutic targets (Zhao et al.). A Mendelian randomization study also showed that genetically predicted serum metabolites mediate associations between inflammatory proteins and PCOS risk, indicating that metabolic intermediates may serve as actionable links between inflammation and disease expression (Jia et al.). One study provided direct translational evidence for immune-based mechanisms: the FertIL trial testing IL-1 receptor antagonism provided early clinical data on immune-modulating strategies in PCOS (Wälchli-Popovic et al.).

## Co-morbidities with PCOS

This Research Topic further explored several aspects of co-morbidities associated with PCOS. ApoB-driven atherogenic dyslipidemia and early subclinical cardiac remodeling were observed in high-risk young women with PCOS. Thus, early lipid and vascular monitoring might be critical long before overt disease manifests (Wu et al.). Disrupted circadian rhythms were systematically linked to PCOS pathogenesis, potentially exacerbating endocrine and metabolic dysfunction (Li et al.). An elevated prevalence of obstructive sleep apnea in PCOS further corroborated sleep disturbances in women with PCOS, highlighting sleep patterns as an essential component of comprehensive care, since sleep pathology can amplify metabolic and cardiovascular risk (Jafar et al.). Sleep disturbance also leads to lower quality of life and mood disturbances. Early psychological symptoms in peri-menarcheal cohorts were associated with later PCOS risk factors, suggesting adolescence might be a critical window for psychosocial screening and early behavioral or mental-health interventions that could alter long-term reproductive and metabolic trajectories (Brink et al.).

## Molecular biomarkers

Finally, new actionable molecules are urgently needed for biomarker development and drug discovery. Accordingly, a bioinformatics analysis mapped hub genes, and mendelian randomization further identified causal molecular relationships in PCOS (He et al.). Experimental validation also identified key oxidative-stress genes implicated in PCOS pathogenesis (Li et al.), providing one potential avenue for candidate molecular target discovery and biomarker development (Li et al.), (He et al.).

Taken together, this Research Topic brings together a range of articles that shed light on different dimensions of PCOS, from metabolic dysfunction to psychological impact, including understudied areas such as sleep disturbances. The articles represent a small step toward building understanding of a condition that remains both common and complex. This is also a call for larger, more diverse studies to capture the full spectrum of disorder manifestations and long−term health outcomes. Individualized treatment approaches, including molecular targets are needed to ensure that care addresses the whole person, not just one aspect of the syndrome.

